# Seasonal Variation in Population Abundance and Chytrid Infection in Stream-Dwelling Frogs of the Brazilian Atlantic Forest

**DOI:** 10.1371/journal.pone.0130554

**Published:** 2015-07-10

**Authors:** Joice Ruggeri, Ana V. Longo, Marília P. Gaiarsa, Laura R. V. Alencar, Carolina Lambertini, Domingos S. Leite, Sergio P. Carvalho-e-Silva, Kelly R. Zamudio, Luís Felipe Toledo, Marcio Martins

**Affiliations:** 1 Instituto de Biologia, Universidade Federal do Rio de Janeiro, Ilha do Fundão, Rio de Janeiro, Brazil; 2 Department of Ecology and Evolutionary Biology, Cornell University, Ithaca, New York, United States of America; 3 Departamento de Ecologia, Instituto de Biociências, Universidade de São Paulo, São Paulo, Brazil; 4 Laboratório de História Natural de Anfíbios Brasileiros (LaHNAB), Departamento de Biologia Animal, Instituto de Biologia, Universidade Estadual de Campinas, Campinas, São Paulo, Brazil; 5 Laboratório de Antígenos Bacterianos, Departamento de Genética, Evolução e Bioagentes, Instituto de Biologia, Universidade Estadual de Campinas, Campinas, São Paulo, Brazil; University of South Dakota, UNITED STATES

## Abstract

Enigmatic amphibian declines were first reported in southern and southeastern Brazil in the late 1980s and included several species of stream-dwelling anurans (families Hylodidae and Cycloramphidae). At that time, we were unaware of the amphibian-killing fungus *Batrachochytrium dendrobatidis* (*Bd*); therefore, pollution, habitat loss, fragmentation and unusual climatic events were hypothesized as primary causes of these declines. We now know that multiple lineages of *Bd* have infected amphibians of the Brazilian Atlantic forest for over a century, yet declines have not been associated specifically with *Bd* outbreaks. Because stream-dwelling anurans occupy an environmental hotspot ideal for disease transmission, we investigated temporal variation in population and infection dynamics of three stream-adapted species (*Hylodes asper*, *H*. *phyllodes*, and *Cycloramphus boraceiensis*) on the northern coast of São Paulo state, Brazil. We surveyed standardized transects along streams for four years, and show that fluctuations in the number of frogs correlate with specific climatic variables that also increase the likelihood of *Bd* infections. In addition, we found that *Bd* infection probability in *C*. *boraceiensis*, a nocturnal species, was significantly higher than in *Hylodes* spp., which are diurnal, suggesting that the nocturnal activity may either facilitate *Bd* zoospore transmission or increase susceptibility of hosts. Our findings indicate that, despite long-term persistence of *Bd* in Brazil, some hosts persist with seasonally variable infections, and thus future persistence in the face of climate change will depend on the relative effect of those changes on frog recruitment and pathogen proliferation.

## Introduction

Enigmatic amphibian declines were first reported in Brazil in the late 1980s and were attributed to unusual climatic events, pollution, habitat loss, and fragmentation [1,2,3,4]. Reported declines included populations of obligate stream-dwelling frogs in the genera *Hylodes*, *Crossodactylus* (Hylodidae), and *Cycloramphus* (Cycloramphidae) throughout the mountainous regions of the Atlantic coastal forest of southern and southeastern Brazil [1,2,3]. At that time, the exact drivers of decline were unknown, and the changes in species abundances were considered enigmatic, yet infectious diseases were proposed as a possible cause because amphibian populations disappeared from relatively undisturbed habitats [2,4].

The pathogenic chytrid fungus *Batrachochytrium dendrobatidis* (hereafter *Bd*) has been implicated in declines of wild amphibian populations worldwide [5,6,7,8], including in pristine areas with high natural vegetation cover [9]. In cases where the pathogen has invaded new areas, susceptible species are extirpated or decline just a few months after *Bd* invasion [10,11,12,13], with highly deleterious effects on naïve host communities [14]. In contrast, retrospective studies have identified localities with long-term *Bd* endemism [15,16]. One of these, the Brazilian Atlantic forest, has the oldest *Bd* infection records (late 1800s) [16] and a high diversity of genetically distinct *Bd* lineages [17,18]. Therefore, Atlantic forest anurans have experienced long-term co-existence with this pathogen, which might explain high infection tolerance in many current anuran populations [19,20].

More than 40% of *Bd*-infected species from southern and southeastern Brazil belong to the families Cycloramphidae and Hylodidae [16]. The genera *Cycloramphus* and *Hylodes* are obligate stream-dwelling frogs, often highly philopatric [21], and typically rely on undisturbed forested habitat [22,23]. Undisturbed forests provide optimal microclimatic conditions for *Bd* proliferation and harbor a greater diversity of host species, enhancing the potential for pathogen transmission [9,24,25,26]. After *Bd* emergence, stream-associated riparian amphibians in naïve communities were the first to show rapid declines [11,27]; thus, their preferred microhabitat has an impact on the probability of *Bd* infection because specific environmental factors in those habitats are more conducive to transmission and pathogen proliferation [9,20,24]. Interestingly, among the Brazilian stream dwelling species in which *Bd* was detected in recent years [19,20], apparently none have shown dramatic population declines, despite relatively high infection prevalence and intensity. Given that the extinction threat for stream-dwellers may be highly underestimated [24], studies evaluating amphibian population dynamics and the impact of *Bd* are a priority for the conservation of these Atlantic forest taxa.

In this study we investigated population dynamics and seasonal changes in *Bd* prevalence in three species of stream-dwelling frogs in the Cycloramphidae and Hylodidae families (*Hylodes asper*, *H*. *phyllodes*, and *Cycloramphus boraceiensis*) on the northern coast of São Paulo state, southeastern Brazil. We surveyed standardized transects along Atlantic forest streams during four years and tested for a correlation between fluctuations in the number of frogs with specific climatic variables that may increase the likelihood of infections by *Bd*. We also quantified *Bd* prevalence and infection intensity in host species during a cool period of low precipitation (winter 2007) and a warmer period of high precipitation (summer 2008). We hypothesized that environmental variables influence both pathogen and host dynamics over seasons in the Atlantic forest, enabling host-pathogen coexistence over the years [16].

## Methods

### Field Sampling

To quantify amphibian population trends over time, we conducted standardized transect surveys in streams in Parque Estadual da Serra do Mar (PESM), which runs along 270 km (from 24°13'12.49" S, 47°22'4.71" W to 23°22'36.90" S, 44°44'19.07" W) of the coast of São Paulo state, southeastern Brazil. Our study area is located in PESM Núcleo Picinguaba, in the municipality of Ubatuba. Fieldwork was approved by Permit #16593–1 issued by MMA, IBAMA, and ICMBio. These Agencies also approved all methods used for collecting and manipulating biological samples. Approval by an Animal Ethics Committee was not required for this study. Approximately 40 species of anurans occur in Núcleo Picinguaba [27,28]. The climate in this region is seasonally wet with mean annual rainfall of 2519 mm, a warmer season from October to April (monthly rainfall 215–376 mm, with peak in December and January; mean temperatures from 21.1–25.5), and a drier, colder season from May to September (monthly rainfall 11–166 mm, with July the driest month; mean temperatures from 18.4–20.5) [29].

We surveyed four low elevation streams (150–330 m asl) on the escarpments of the Serra do Mar located along 4 km of the Rio-Santos highway (S1 Table). These four streams were chosen to represent the variation in width, slope, and water flow typical of small streams in the study area. Monthly surveys were conducted from February 2007 to January 2011. In each stream we sampled a 100–120 m long transect. We searched for adults of the three focal species of stream-dwelling frogs (*H*. *asper*, *H*. *phyllodes*, and *C*. *boraceiensis*) by slowly walking upstream once during daytime and once at night for 30–90 minutes.

### Environmental data

Maximum and minimum temperature and total precipitation were obtained from the Ubatuba weather station (INMET) [30]. This station is located 28 km from the study site; based on the relative homogeneity of the climate in the region, we assume that the climate at the study site is similar to that in Ubatuba.

### *Bd* sampling and quantification

To quantify seasonal patterns in *Bd* infection prevalence and intensity, we swabbed 20 frogs of each species once during winter (August 2007) and once during summer (February 2008). We captured frogs using non-powdered latex gloves to avoid transmission between animals and sample contamination, and followed standard field sampling protocols [31,32]. Each swab was stored in sterile plastic tubes, at -20°C, and frogs were immediately released at the site of capture. To quantify the presence and infection intensity of *Bd* in each sample, we first extracted DNA from swabs using PrepMan ULTRA (Life Technologies), and then quantified infection intensities using a Taqman qPCR Assay (Life Technologies) with standards of 0.1, 1, 10, 100, and 1000 zoospore genomic equivalents (g.e.) [31,33] using *Bd* strain CLFT023. We considered a sample as “*Bd-*positive” when the infection load was greater than or equal to one g.e. [31]. DNA samples are deposited in the SLFT Collection, at the Universidade Estadual de Campinas (S2 Table).

To evaluate the prevalence of *Bd* in each population, we followed the protocol where the number of sampled frogs is equal to 3/p (prevalence expressed in proportion) [34], except for the population of *H*. *asper* in summer 2008.

### Statistical analyses

We performed seasonal-trend decomposition analyses to the monthly estimates of number of individuals found per 100 m and the monthly climatic data (min. temperature and precipitation). We pooled the data collected on the four stream transects and used the mean number of individuals per species to create time series objects in R [35]. Each time series was decomposed into its seasonal components using a periodic loess window [36], in which seasonal values are removed and remaining values are smoothed to detect the underlying trend [36]. In addition, we tested the temporal stability of populations by fitting separate general linear models (GLM) for each species. In these models, our independent variable was the average number of individuals. Calendar year, minimum and maximum temperature, and precipitation were fixed factors, while month and calendar year were nested and set as random factors to control for natural monthly fluctuations within each year.

We tested for seasonality in *Bd* prevalence and infection intensity with generalized linear models (GLMs) using logistic and quasipoisson regressions. Our logistic model for *Bd* presence/absence included season, species, and their interaction as predictors. Our quasipoisson model for *Bd* infection intensity included the same predictor variables. All statistical models were implemented in R [35].

## Results

### Environmental data

During the four-year monitoring period the climate in the study area became warmer and drier, with mean temperatures varying from 23–25°C in the first years to 27°C in 2010, and precipitation decreasing from 52 mm in 2007 to 29 mm in the last year of the study.

### Population dynamics

**Fig 1:**
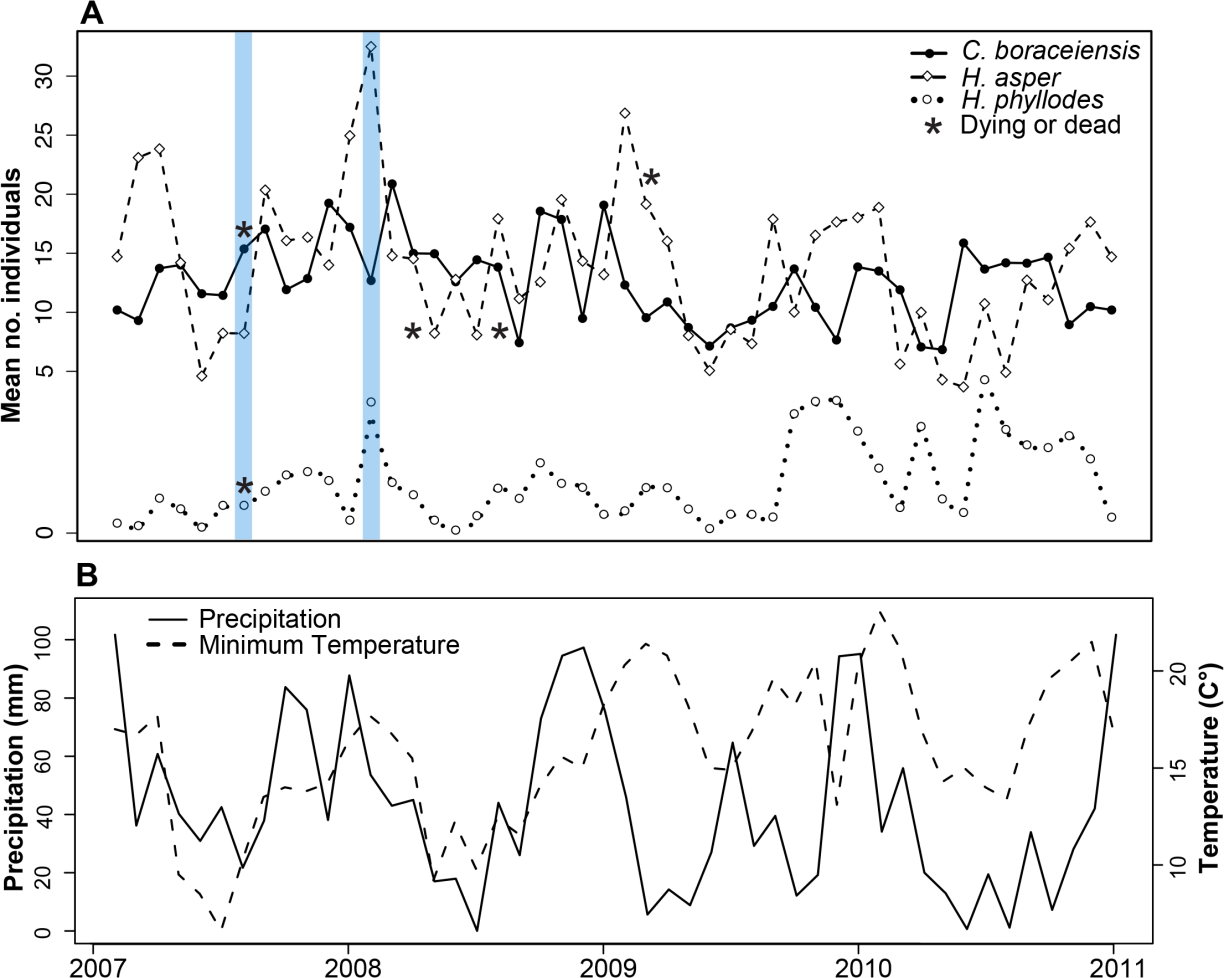
Mean number of individuals, temperature and precipitation. Four-year time series data of: (A) the mean number of individuals found in four stream transects, and (B) monthly minimum temperature (°C) and precipitation (mm) at Parque Estadual da Serra do Mar, Brazil. Shaded bars highlight *Batrachochytrium dendrobatidis* swab sampling. The first asterisk (dead or dying frog) for *C*. *boraceiensis* represents two individuals found within 3 days. Sampling began February 2007 and continued monthly through January 2011.

**Fig 2:**
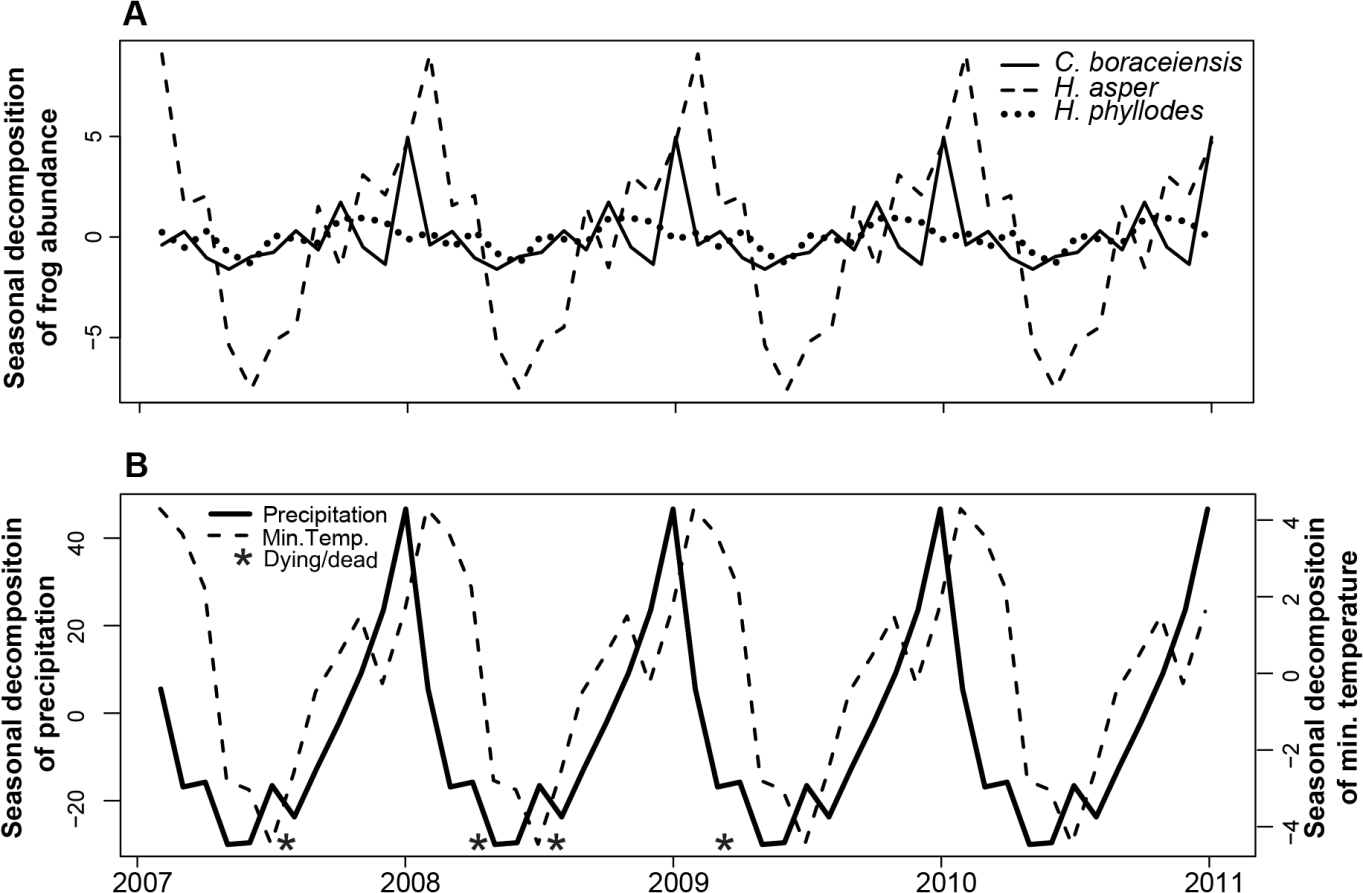
Seasonal decomposition of frog abundance, temperature and precipitation. Seasonal decomposition of the time series data of: (A) the mean number of individuals found in four stream transects, and (B) monthly minimum temperature (°C) and precipitation (mm) at Parque Estadual da Serra do Mar, Brazil.

Our surveys showed high variability in the average number of individuals for each of our three focal species, but also common peaks in frog abundance during January-March (warm season) and declines during the months of June-July (cold season) (Fig 1). *Hylodes asper* was the most common species at our sites with an average abundance (± standard deviation) of 14.0 ± 6.3 individuals per survey, followed by *C*. *boraceiensis* with 12.8 ± 3.7 individuals, and *H*. *phyllodes* with 1.5 ± 1.2 individuals (Fig 1). Fluctuations in the number of individuals corresponded to seasonal patterns in temperature and precipitation (Fig 1B). Time series decomposition of frog abundances and climatic variables helped us distinguish seasonal components and the separate cycles of high temperature and precipitation from cycles of cooler temperatures and drier conditions (Fig 2) that likely cause changes in amphibian density. Despite these cycles, minimum temperature only predicted abundance changes in *H*. *asper* (GLM, *t*_1, 28_ = 5.01, *P* < 0.001).

**Fig 3:**
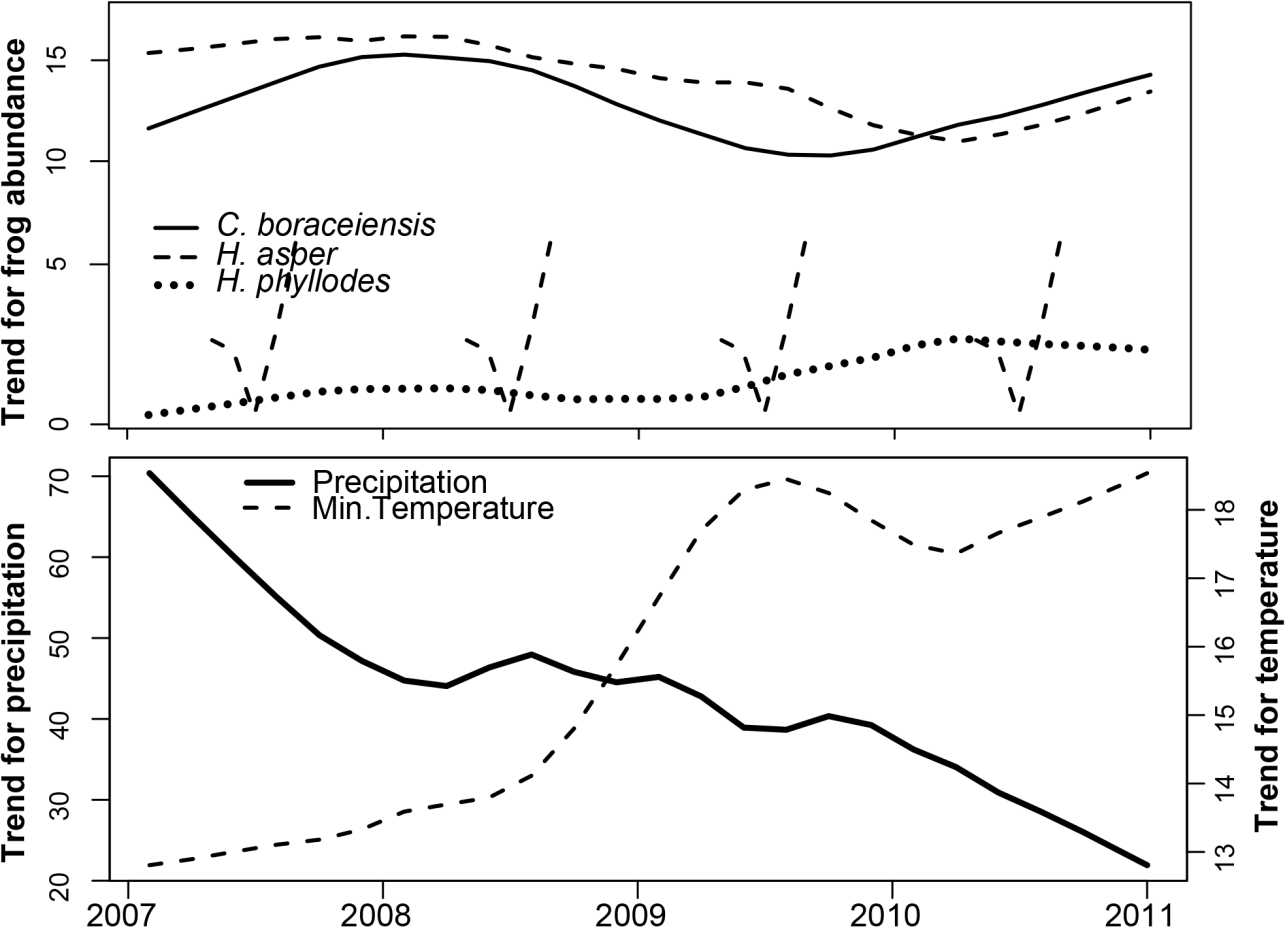
Frog abundance, temperature and precipitation. Trends of the time series data of: (A) the mean number of individuals found in four stream transects, and (B) monthly minimum temperature (°C) and precipitation (mm) at Parque Estadual da Serra do Mar, Brazil.

Our seasonal-trend decomposition analyses revealed apparent slight population declines of *H*. *asper* and *C*. *boraceiensis* in mid-2008 that extended through 2009 (Fig 3A). In contrast, *H*. *phyllodes* populations exhibited a slight increase in the first months of 2009 (Fig 3A). These changes were concordant with an increase in minimum temperature and decrease in precipitation at the end of 2008 (Fig 3B). Our linear mixed-effects models confirmed declines in abundance of *H*. *asper* during 2009 (GLM, *t*_1, 28_ = −2.89, *P* < 0.01) and 2010 (GLM, *t*_1, 28_ = −4.11, *P* < 0.001), as well as the increase in abundance for *H*. *phyllodes* in 2010 (GLM, *t*_1, 28_ = 2.33, *P* = 0.027).

### *Bd* infection dynamics

**Fig 4:**
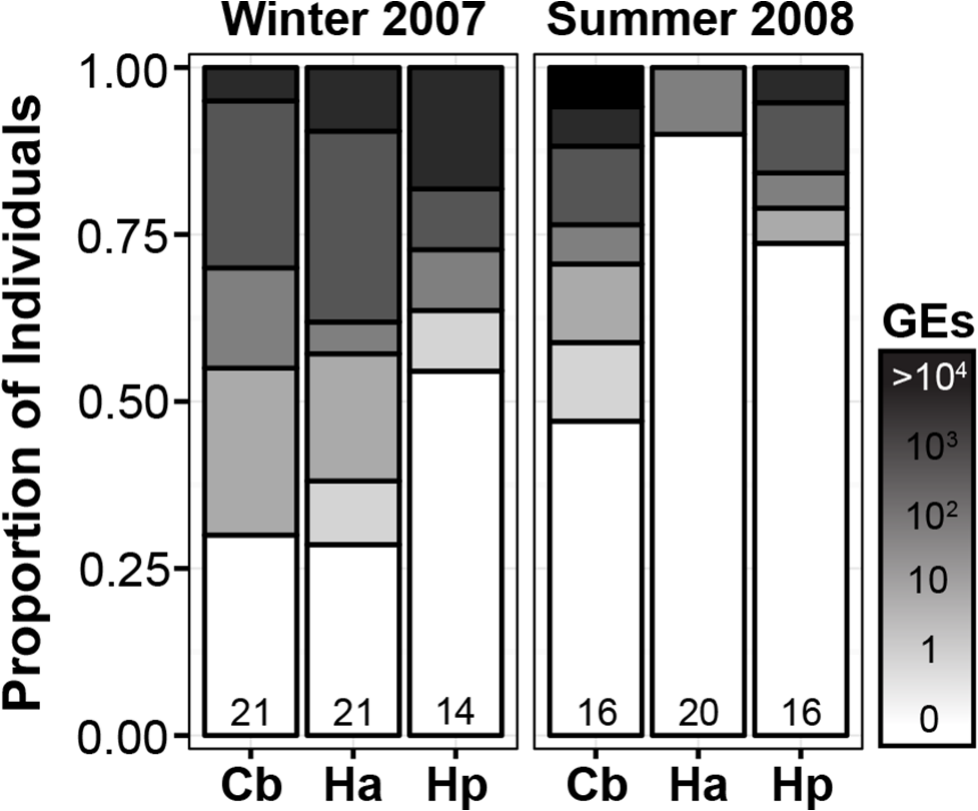
Intensity of *Bd* infection. *Batrachochytrium dendrobatidis* infection intensity of *Cycloramphus boraceiensis* (Cb), *Hylodes asper* (Ha) and *Hylodes phyllodes* (Hp) during winter and summer stream surveys at Parque Estadual da Serra do Mar, Brazil. The total number of individuals swabbed is shown in the bottom of each bar. We measured genomic equivalents (GEs) using qPCR, and report the proportion of individuals infected by each zoospore category: 0, 1–10, 11–100, 101–1000, and >1001 GEs.

Our logistic model of *Bd* infection revealed significant effects of season (*β* = 3.11 ± 0.89, *DF* = 102, *Z* = 3.51, *P* < 0.001) and species (*β* = 2.20 ± 0.90, *DF* = 102, *Z* = 2.45, *P* = 0.014). The prevalence of *Bd* in individuals sampled during winter was significantly higher than those sampled during summer (Fig 4) (*P* < 0.001), and *C*. *boraceiensis* showed higher infection load than the other two species. In addition, we found a marginal effect of the interaction between season and species for *C*. *boraceiensis* (*β* = -2.20 ± 1.13, *DF* = 102, *Z* = -1.95, *P* = 0.051). We did not detect any effects of these variables on *Bd* infection intensity, even after excluding uninfected frogs (Quasipoisson GLM: all *P* > 0.05).

We observed six cases of dead or dying frogs in the field during the course of the demographic study (three *H*. *asper*, two *C*. *boraceiensis*, and one *H*. *phyllodes*; Fig 1A, represented by asterisks). Frog mortality occurred mostly during the cool-dry periods of the year (Fig 2B).

The relationship between seasonal variation in *Bd* prevalence and host abundance was different across the three species. *Cycloramphus boraceiensis* showed no seasonal pattern of infection and population abundance. The prevalence of *Bd* in the population of *Hyllodes phyllodes* was higher during winter (*P* = 0.05) despite its lower population abundance (Fig 1A). In contrast, *Hylodes asper* showed different *Bd* prevalence between seasons (*P* < 0.001), being more infected during the winter than summer, when the number of individuals was also greater (Fig 1A). Although infection in the population of *H*. *asper* may have been underestimated during summer 2008 due to smaller sample size, these results indicate that seasonality may have different effects on disease dynamics across syntopic species. Re-analyses with larger sample sizes of population disease data would be interesting to confirm these possible differences.

## Discussion

Understanding the effects of pathogens on wildlife requires long-term datasets to identify potential changes in natural population dynamics [37,38]. Our demographic surveys indicate that the three focal species of stream-dwelling frogs in Brazil have experienced periods of slight population growth, declines, and recovery over the four-year study. At the same time, climate in this area has become warmer and drier. Although we did not include a continuous survey of *Bd* infection through time for these three species, we found a higher probability of infection during winter months, and we report *Bd*-associated mortalities during cool-dry periods, which have also been observed in other studies of *Bd* enzootics in the tropics [39–41].

Our three focal species live in the same microhabitat, thus the risk of exposure and infection is likely constant across species, yet our *Bd* survey data indicate that the three species rely on different strategies to persist under climate variation and disease. *Hylodes* species are diurnal; in contrast, *Cycloramphus* species are crepuscular and nocturnal [23]. Temperatures differ between diurnal and nocturnal activity periods, and this in turn potentially modulates immune responses to infection [42] or the ability of hosts to reduce infection loads through behavioral fever [43,44]. Therefore, diurnal behavior may benefit *Hylodes* spp. by providing opportunities to increase their body temperature above 30°C, a temperature that kills infective zoospores [44,45]. Indeed, we observed individuals of both species of *Hylodes* under direct sunlight in many instances. In contrast, *C*. *boraceiensis* retreat in rock crevices during the day and do not experience the same breadth in temperatures, and we never found *C*. *boraceiensis* under direct sunlight [46]. Additionally, susceptibility to *Bd* infection may differ between the frog lineages studied (Hylodidae and Cycloramphidae) due to variation in immune function [47,48]. Future studies should investigate the immune limitations imposed by strictly nocturnal behavior as well as a possible phylogenetic signal in the vulnerability to *Bd* infection.

Long-term monitoring is especially important in cases of hosts surviving with enzootic pathogens, because only temporal demographic patterns can reveal if the observed changes in population size are of a scale that negatively impacts the probability of long-term population persistence. Fluctuations in environmental variables can affect the stability of amphibian population dynamics by influencing species’ survival, fecundity, breeding, or recruitment [49]. Only one previous study has estimated population parameters for our focal species. In *H*. *asper*, recruitment of juveniles is high and populations have almost complete annual turnover, raising concerns that even minor environmental changes could affect populations [50], and perhaps explain local extinctions of *Hylodes* observed in the late 1970s [1]. Indeed, our monitoring studies provide additional support for these findings by showing a decline in mid-2008 that corresponded with an increase in temperature and drier conditions (Fig 3). At the population level, our findings indicate that both *H*. *phyllodes* and *C*. *boraceiensis* showed a decrease in *Bd* prevalence over a period of six months (20–30% reduction in prevalence, Fig 4). Combined, our results show how two different stressors (infection and climate variation) can interact to affect population dynamics. Fortunately, the three species studied here show apparent resilience to these changes in temperature, precipitation, and infection. However, whether or not this advantage carried sub-lethal fitness costs is unknown.

In addition to revealing interspecific differences in the probability of infection among stream-dwelling frogs, our results corroborate seasonal variation in the prevalence of *Bd*, a pattern that has been documented in other tropical and temperate taxa [40,51,52]. In general, we found more infected frogs in the month with the coolest air temperature (Fig 4), indicating that environmental variables potentially increase host vulnerability to *Bd* in cold temperatures by one of two mechanisms: (1) immunosuppression or (2) stimulating pathogen replication in the environment. In fact, although cooler temperatures decrease *Bd* growth rate, they also stimulate zoosporangia to produce greater number of zoospores, which remain infectious for longer periods, a life history trade-off that could favor *Bd* transmission at lower temperatures [53].

According to the Intergovernmental Panel on Climate Change [54], many areas in the Americas will experience a 4–4.5°C increase in surface temperatures and severe droughts as the result of anthropogenic climate change [54]. These projections also forecast an increase of 10–15% in autumn rainfall and increased droughts during the austral summer [54]. These changes impact frog phenology in different ways. First, more rainfall during autumn will increase reproduction in active frogs, but could limit juvenile survival if earlier life stages are more vulnerable to winter infection [55,56]. Second, droughts increase stress in amphibians and may also limit juvenile recruitment if species are not able to adapt rapidly to these novel conditions [57,58]. Alternatively, given the resilience observed in these three species, we can also predict a scenario more favorable for amphibians, in which increased temperatures could benefit temperature-dependent immune responses and the capacity to express behavioral fever. This would require very specific physiological adaptations to temperature as well as the availability of different microhabitats for thermoregulation. However, due to the tight interplay between hosts and pathogen, these scenarios will likely depend on the responses of the pathogen, which could be highly plastic [59]. Future studies in seasonally variable regions where *Bd* is endemic should focus on understanding the evolutionary potential of thermal tolerance across *Bd* lineages, as well as quantifying juvenile survival and recruitment under different temperatures and infection scenarios.

## Supporting Information

S1 Table Geographic positions and brief description of each stream transect. (PDF)

S2 Table Data from amphibian samples collected at Parque Estadual da Serra do Mar, Picinguaba. (PDF)

## References

[1] Heyer WR, Rand AS, Cruz CAG, Peixoto OL. Decimations, extinctions, and colonizations of frog populations in Southeast Brazil and their evolutionary implications. Biotropica. 1988;20: 230–235.

[2] Weygoldt P. Changes in the composition of mountain stream frog communities in the Atlantic mountains of Brazil: frogs as indicators of environmental deteriorations? Stud Neotrop Fauna Environ. 1989;243: 249–255.

[3] Eterovick PC, Carnaval ACOQ, Borges-Nojosa DM, Silvano DL, Segalla MV, Sazima I. Amphibian declines in Brazil: an overview. Biotropica. 2005; 37: 166–179.

[4] Guix JC, Montori A, Llorente GA, Carretero MA, Santos X. Natural history of Bufonids in four Atlantic Rainforest areas of the Southeastern Brazil. Herpet Nat His. 1998;6: 1–12.

[5] Longcore JE, Pessier AP, Nichols DK. *Batrachochytrium dendrobatidis* gen. et sp. nov., a chytrid pathogenic to amphibians. Mycol. 1999;91: 219–227.

[6] Collins JP, Storfer A. Global amphibian declines: sorting the hypotheses. Divers Distrib. 2003;9: 89–98.

[7] Puschendorf R, Bolaños F, Chaves G. The amphibian chytrid fungus along an altitudinal transect before the first reported declines in Costa Rica. Biol Conserv. 2006;132: 136–142.

[8] Catenazzi A, von May R, Vredenburg VT. High prevalence of infection in tadpoles increases vulnerability to fungal pathogen in high-Andean amphibians. Biol Conserv. 2013;159: 413–421.

[9] Becker CG, Zamudio KR. Tropical amphibian populations experience higher disease risk in natural habitats. PNAS. 2011;18 **:** 9893–9898. 10.1073/pnas.1014497108 PMC3116417 21628560

[10] Crawford AJ, Lips KR, Bermingham E. Epidemic disease decimates amphibian abundance, species diversity, and evolutionary history in the highlands of central Panama. PNAS. 2010;107: 13777–13782. 10.1073/pnas.0914115107 20643927 PMC2922291

[11] Lips KR, Brem F, Brenes R, Reeve JD, Alford RA, Voyles J, et al Emerging infectious disease and the loss of biodiversity in a neotropical amphibian community. PNAS. 2006;103:3165–3170. 16481617 10.1073/pnas.0506889103 PMC1413869

[12] Lips KR, Diffendorfer J, Mendelson JR III, Sears MW. Riding the Wave: Reconciling the Roles of Disease and Climate Change in Amphibian Declines. PLoS Biol. 2008;6: 441–454. 10.1371/journal.pbio.0060072 PMC2270328 18366257

[13] Vredenburg VT, Knapp RA, Briggs CJ. Dynamics of an emerging disease drive large-scale amphibian population extictions. PNAS. 2010;107: 9689–9694. 10.1073/pnas.0914111107 20457913 PMC2906868

[14] Ouellet M, Mikaelian I, Pauli BD, Rodrigues J, Green DM. Historical evidence of widespread Chytrid infection in North American Amphibian populations. Conserv Biol. 2005;19: 1431–1440.

[15] Goka K, Yokoyama J, Une Y, Kuroki T, Suzuki K, Nakahara M, et al Amphibian chytridiomycosis in Japan: distribution, haplotypes and possible route of entry into Japan. Mol Ecol. 2009;18: 4757–4774. 10.1111/j.1365-294X.2009.04384.x 19840263

[16] Rodriguez D, Becker CG, Pupin NC, Haddad CFB, Zamudio KR. Long-term endemism of two highly divergent lineages of the amphibian-killing fungus in the Atlantic Forest of Brazil. Mol Ecol. 2014;23: 774–787. 10.1111/mec.12615 24471406

[17] Schloegel LM, Toledo LF, Longcore JE, Greenspan SE, Vieira CA, Lee M, et al Novel, panzootic, and hybrid genotypes of amphibian chytridiomycosis associated with the bullfrog trade. Mol Ecol. 2012;21: 5162–5177. 10.1111/j.1365-294X.2012.05710.x 22857789

[18] Rosenblum EB, James TY, Zamudio KR, Poorten TJ, Ilut D, Rodriguez D, et al Complex history of the amphibian-killing chytrid fungus revealed with genome resequencing data. PNAS. 2013;110: 9385–9390. 10.1073/pnas.1300130110 23650365 PMC3677446

[19] Toledo LF, Haddad CFB, Carnaval AOCQ, Britto FB. A Brazilian anuran (*Hylodes magalhaesi*: Leptodactylidae) infected by *Batrachochytrium dendrobatidis*: a conservation concern. Amphib Reptile Conserv. 2006;4: 17–21.

[20] Gründler MC, Toledo LF, Parra-Olea G, Haddad CFB, Giasson LOM, Sawaya RJ, et al Interaction between breeding habitat and elevation affects prevalence but not infection intensity of *Batrachochytrium dendrobatidis* in Brazilian anuran assemblages. Dis Aquat Org. 2012;97: 173–184. 10.3354/dao02413 22422088

[21] Welsh HH Jr, Ollivier LM. Stream amphibians as indicator s of ecosystem stress: a case study from California’s redwoods. Ecol Appl. 1998;8: 1118–1132.

[22] Haddad CFB, Toledo LF, Prado CPA, Loebmann D, Gasparini JL, Sazima I. Guide to the amphibians of the Atlantic Forest: Diversity and Biology São Paulo, Anolisbook 2013.

[23] Almeida-Gomes M, Lorini ML, Rocha CFD, Vieira MV. Underestimation of extinction threat to stream-dwelling amphibians due to lack of consideration of narrow area of occupancy. Conserv Biol. 2013;28: 616–619. 10.1111/cobi.12196 24372858

[24] McDonald K, Alford R. A Review of Declining Frogs in Northern Queensland. In: Campbell A, editor. Declines and Disappearances of Australian Frogs. National Threatened Frog Workshop; 1999. Pp. 14–22.

[25] Becker CG, Rodriguez D, Longo AV, Talaba AL, Zamudio KR. Disease risk in temperate amphibian populations is higher at closed-canopy sites. PLoS ONE. 2012;7: e48205 10.1371/journal.pone.0048205 23118953 PMC3485156

[26] Becker CG, Rodriguez D, Toledo LF, Longo AV, Lambertini C, Corrêa DT, et al Partitioning the net effect of host diversity on an emerging amphibian pathogen. Proc R Soc B. 2015;281: 20142881. 10.1098/rspb.2014.1796 PMC4213625 25297867

[27] Hartmann MT, Hartmann PA, Haddad CFB. Reproductive modes and fecundity of an assemblage of anuran amphibians in the Atlantic rainforest, Brazil. Iheringia. 2010;100: 207–215. 10.1017/S0007485309990162 19580687

[28] Toledo LF, Becker CG, Haddad CFB, Zamudio KR. Rarity as an indicator of endangerment in Neotropical frogs. Biol Conserv. 2014;179: 54–62.

[29] EMBRAPA (2014) Ministério da Agricultura, Pecuária e Abastecimento. Available: https://www.embrapa.br/.

[30] Agritempo: Sistema de Monitoramento Agrometeorológico (2002) Ministério da Agricultura, Pecuária e Abastecimento. Estação Ubatuba (INMET). Available: http://www.agritempo.gov.br/agritempo/jsp/Estacao/index.jsp?siglaUF=SP.

[31] Kriger KM, Hero JM, Ashton KJ. Cost efficiency in the detection of chytridiomycosis using PCR assay. Dis Aquat Org. 2006;71: 149–154. 16956062 10.3354/dao071149

[32] Hyatt AD, Boyle DG, Olsen V, Berger L, Obendorf D, Dalton A, et al Diagnostic assays and sampling protocols for the detection of *Batrachochytrium dendrobatidis* . Dis Aquat Org. 2007;73: 175–192. 17330737 10.3354/dao073175

[33] Lambertini C, Rodriguez D, Brtio FB, Leite DS, Toledo LF. Diagnóstico do fungo Quitrídio: *Batrachochytrium dendrobatidis* . Herpetol Bras. 2013;2: 12–17.

[34] Skerratt LF, Berger L, Hines HB, McDonald KR, Mendez D, Speare R. Survey protocol for detecting chytridiomycosis in all Australian frog populations. Dis Aquat Org. 2008;80: 85–94. 10.3354/dao01923 18717061

[35] R Development Core Team. R: A Language and Environment for Statistical Computing R Foundation for Statistical Computing 2013.

[36] Cleveland RB, Cleveland WS, McRae JE, Terpenning I. STL: A seasonal-trend decomposition procedure based on LOESS. J Offic Stat. 1990;6: 3–73.

[37] Murray KA, Skerratt LF, Speare R, McCallum HI. Impact and dynamics of disease in species threatened by the Amphibian Chytrid Fungus, *Batrachochytrium dendrobatidis* . Conserv Biol. 2009;23: 1–11. 10.1111/j.1523-1739.2008.01142.x 19774709

[38] Scheele BC, Guarino F, Osborne W, Hunter DA, Skerratt LF, Driscoll DA. Decline and re-expansion of an amphibian with high prevalence of chytrid fungus. Biol Conserv. 2014;170: 86–91.

[39] Longo AV, Ossiboff RJ, Zamudio KR, Burrowes PA. Lability in host defenses: Terrestrial frogs die from Chytridiomycosis under enzootic conditions. J Wildl Dis. 2013;49: 197–199. 10.7589/2012-05-129 23307390

[40] Whitfield SM, Kerby J, Gentry LR, Donnelly MA. Temporal variation in infection prevalence by the Amphibian Chytrid fungus in three species of frogs at La Selva, Costa Rica. Biotropica. 2012;44: 779–784.

[41] Retallick RWR, McCallum H, Speare R. Endemic Infection of the Amphibian Chytrid Fungus in a frog community post-decline. PLoS Biol. 2004;2: 1965–1971. 10.1371/journal.pbio.0020351 PMC521176 15502873

[42] Raffel TR, Rohr JR, Kiesecker JM, Hudson PJ. Negative effects of changing temperature on amphibian immunity under field conditions. Funct Ecol. 2006;20: 819–828.

[43] Richards-Zawacki CL. Thermoregulatory behavior affects prevalence of chytrid fungal infection in a wild population of Panamanian golden frogs. Proc R Biol Sci. 2010;277: 519–528. 10.1098/rspb.2009.1656 19864287 PMC2842693

[44] Rowley JJ, Alford RA. Hot bodies protect amphibians against chytrid infection in nature. Sci Rep. 2013;3:1515 10.1038/srep01515 23519020 PMC3604863

[45] Piotrowski JS, Annis SL, Longcore JE. Physiology of *Batrachochytrium dendrobatidis*, a chytrid pathogen of amphibians. Mycol. 2004;96: 9–15. 21148822

[46] Boltaña S, Rey S, Roher N, Vargas R, Huerta M, Huntingford FA, et al. Behavioural fever is a synergic signal amplifying the innate immune response. Proc R Biol Sci. 2013; 10.1098/rspb.2013.1381 PMC3730603 23843398

[47] Longo AV, Burrowes P, Zamudio KR. Genomic studies of disease-outcome in host-pathogen dynamics. Integ Comp Biol. 2014;54: 427–438. 10.1093/icb/icu073 24916476

[48] Ellison AR, Savage AE, DiRenzo GV, Langhammer P, Lips KR, Zamudio KR. Fighting a losing battle: vigorous immune response countered by pathogen suppression of host defenses in a chytridiomycosis-susceptible frog G3 –Genes Genom Genet. 2014;4: 1275–1289. 10.1534/g3.114.010744 PMC4455776 24841130

[49] Corn PS. Climate change and amphibians. Anim Biodivers Conserv. 2005;28 1: 59–67.

[50] Patto CEG, Pie MR. Notes on the population dynamics of *Hylodes asper* in Southeastren Brazil (Anura: Leptodactylidae). J Herpetol. 2001;35: 684–686.

[51] Longo AV, Burrowes PA, Joglar RL. Seasonality of *Batrachochytrium dendrobatidis* infection in direct-developing frogs suggests a mechanism for persistence. Dis Aquat Org. 2009;92: 253–260. 10.3354/dao02054 21268989

[52] Puschendorf R, Hoskin CJ, Cashins SD, McDonald K, Skerratt LF, Vanderwal J, et al Environmental refuge from disease-driven Amphibian Extinction. Conserv Biol. 2011;25: 956–964. 10.1111/j.1523-1739.2011.01728.x 21902719

[53] Woodhams DC, Alford RA, Briggs CJ, Johnson M, Rollins-Smith LA. Life-history trade-offs influence disease in changing climates: strategies of an amphibian pathogen. Ecol. 2008;89: 1627–1639. 18589527 10.1890/06-1842.1

[54] Stocker TF, Qin D, Plattner G-K, Tignor M, Allen SK, Boschung J, et al Climate Change 2013: The Physical Science Basis Contribution of Working Group I to the Fifth Assessment Report of the Intergovernmental Panel on Climate Change. Cambridge and New York: Cambridge University Press 2013.

[55] Lamirande EW, Nichols DK. Effects of host age on susceptibility to cutaneous chytridiomycosis in blue and yellow poison dart frogs (Dendrobates tinctorius) In McKinnell RG, Carlson DL editors. Proceedings of the Sixth International Symposium on the Pathology of Reptiles and Amphibians. Saint Paul: Minnesota; 2002 pp. 3–13.

[56] Kriger MK, Hero J. Altitudinal distribution of chytrid (*Batrachochytrium dendrobatidis*) infection in subtropical Australian frogs. Austral Ecol. 2008;33: 1022–1032.

[57] Scheele BC, Driscoll DA, Fischer J, Hunter DA. Decline of an endangered amphibian during an extreme climatic event. Ecosphere. 2012;3: 1–15.

[58] Walls SC, Barichivich WJ, Brown ME. Drought, Deluge and Declines: The Impact of Precipitation Extremes on Amphibians in a Changing Climate. Biology. 2013; 10.3390/biology2010399 PMC4009861 24832668

[59] Woodhams DC, Alford RA, Briggs CJ, Johnson M, Rollins-Smith LA. Life history trade-offs influence disease in changing climates: strategies of an amphibian pathogen. Ecology. 2008;89: 1627–1639. 18589527 10.1890/06-1842.1

