# Supplementary material for: Seasonal Variation in Population Abundance and Chytrid Infection in Stream-Dwelling Frogs of the Brazilian Atlantic Forest
Source: PLoS One. 2015 Jul 10;10(7):e0130554. doi: 10.1371/journal.pone.0130554 (PMC4498819; doi:10.1371/journal.pone.0130554)
Supplement: S1 Table — (PDF) [file pone.0130554.s001.pdf]

Table S1. Geographic positions and brief description of each stream transect.

Transect Name	Coordinates	Stream description
ST1	23° 21' 53.7" S, 44° 48' 2.8" W	5-10m width, steep slope first 120m, many waterfalls and large pools.
ST2	23° 21' 41.2" S, 44° 47' 15.3" W	5-10m width, low inclination, few waterfalls and many large pools.
ST3	23° 21' 34.4" S, 44° 47' 3.2" W	1-5 m, relatively flat first 40m, and then steep slope. Intermittent stream.
ST4	23° 21' 15.2" S, 44° 46' 3.2" W	4-15m, relatively flat, few waterfalls and many large pools.
