# Supplementary material for: Seasonal Variation in Population Abundance and Chytrid Infection in Stream-Dwelling Frogs of the Brazilian Atlantic Forest
Source: PLoS One. 2015 Jul 10;10(7):e0130554. doi: 10.1371/journal.pone.0130554 (PMC4498819; doi:10.1371/journal.pone.0130554)
Supplement: S2 Table — (PDF) [file pone.0130554.s002.pdf]

Sample	Zoospore g.e.	Family	Genus	Species
SLFT 339	0.85	Hylodidae	*Hylodes*	*asper*
SLFT 340	0.00	Hylodidae	*Hylodes*	*asper*
SLFT 341	0.25	Hylodidae	*Hylodes*	*asper*
SLFT 342	0.00	Hylodidae	*Hylodes*	*asper*
SLFT 343	5.72	Hylodidae	*Hylodes*	*asper*
SLFT 344	8.71	Hylodidae	*Hylodes*	*asper*
SLFT 345	0.00	Hylodidae	*Hylodes*	*asper*
SLFT 346	6.66	Hylodidae	*Hylodes*	*asper*
SLFT 347	199.82	Hylodidae	*Hylodes*	*asper*
SLFT 348	0.00	Hylodidae	*Hylodes*	*asper*
SLFT 349	10.31	Hylodidae	*Hylodes*	*asper*
SLFT 350	4.85	Cycloramphidae	*Cycloramphus*	*boraceiensis*
SLFT 351	4.06	Cycloramphidae	*Cycloramphus*	*boraceiensis*
SLFT 352	21.47	Cycloramphidae	*Cycloramphus*	*boraceiensis*
SLFT 353	0.00	Cycloramphidae	*Cycloramphus*	*boraceiensis*
SLFT 354	0.00	Cycloramphidae	*Cycloramphus*	*boraceiensis*
SLFT 355	0.00	Cycloramphidae	*Cycloramphus*	*boraceiensis*
SLFT 356	3.27	Cycloramphidae	*Cycloramphus*	*boraceiensis*
SLFT 357	0.00	Cycloramphidae	*Cycloramphus*	*boraceiensis*
SLFT 358	107.22	Cycloramphidae	*Cycloramphus*	*boraceiensis*
SLFT 359	10.97	Cycloramphidae	*Cycloramphus*	*boraceiensis*
SLFT 360	3.99	Cycloramphidae	*Cycloramphus*	*boraceiensis*
SLFT 361	0.00	Cycloramphidae	*Cycloramphus*	*boraceiensis*
SLFT 362	0.00	Cycloramphidae	*Cycloramphus*	*boraceiensis*
SLFT 363	11.81	Cycloramphidae	*Cycloramphus*	*boraceiensis*
SLFT 364	0.34	Hylodidae	*Hylodes*	*phyllodes*
SLFT 365	2,721.92	Cycloramphidae	*Cycloramphus*	*boraceiensis*
SLFT 366	958.86	Hylodidae	*Hylodes*	*asper*
SLFT 367	86.54	Hylodidae	*Hylodes*	*asper*
SLFT 368	80.62	Cycloramphidae	*Cycloramphus*	*boraceiensis*
SLFT 369	889.92	Cycloramphidae	*Cycloramphus*	*boraceiensis*
SLFT 370	187.82	Cycloramphidae	*Cycloramphus*	*boraceiensis*
SLFT 371	9,578.54	Hylodidae	*Hylodes*	*asper*
SLFT 372	143.46	Cycloramphidae	*Cycloramphus*	*boraceiensis*
SLFT 373	580.40	Cycloramphidae	*Cycloramphus*	*boraceiensis*
SLFT 374	1,600.90	Hylodidae	*Hylodes*	*phyllodes*
SLFT 375	0.00	Hylodidae	*Hylodes*	*phyllodes*
SLFT 376	0.00	Hylodidae	*Hylodes*	*phyllodes*
SLFT 377	567.36	Hylodidae	*Hylodes*	*phyllodes*
SLFT 378	0.00	Hylodidae	*Hylodes*	*phyllodes*
SLFT 379	0.00	Hylodidae	*Hylodes*	*phyllodes*
SLFT 380	202.55	Hylodidae	*Hylodes*	*asper*
SLFT 381	516.23	Hylodidae	*Hylodes*	*asper*
SLFT 382	826.60	Hylodidae	*Hylodes*	*asper*
SLFT 383	6,247.29	Hylodidae	*Hylodes*	*phyllodes*
SLFT 384	74.56	Hylodidae	*Hylodes*	*phyllodes*
SLFT 385	519.30	Hylodidae	*Hylodes*	*asper*

SLFT 386	0.00	Hylodidae	*Hylodes*	*asper*
SLFT 387	1,059.75	Hylodidae	*Hylodes*	*asper*
SLFT 388	0.00	Hylodidae	*Hylodes*	*asper*
SLFT 389	0.00	Hylodidae	*Hylodes*	*phyllodes*
SLFT 390	0.00	Hylodidae	*Hylodes*	*phyllodes*
SLFT 391	0.00	Hylodidae	*Hylodes*	*phyllodes*
SLFT 392	862.02	Hylodidae	*Hylodes*	*phyllodes*
SLFT 393	77.46	Hylodidae	*Hylodes*	*phyllodes*
SLFT 394	16,883.62	Cycloramphidae	*Cycloramphus*	*boraceiensis*
SLFT 395	0.00	Hylodidae	*Hylodes*	*asper*
SLFT 396	0.00	Hylodidae	*Hylodes*	*asper*
SLFT 397	0.00	Hylodidae	*Hylodes*	*asper*
SLFT 398	0.00	Hylodidae	*Hylodes*	*asper*
SLFT 399	0.00	Hylodidae	*Hylodes*	*asper*
SLFT 400	0.00	Hylodidae	*Hylodes*	*asper*
SLFT 401	95.41	Hylodidae	*Hylodes*	*asper*
SLFT 402	0.00	Hylodidae	*Hylodes*	*asper*
SLFT 403	0.00	Hylodidae	*Hylodes*	*asper*
SLFT 404	0.00	Cycloramphidae	*Cycloramphus*	*boraceiensis*
SLFT 405	105.27	Cycloramphidae	*Cycloramphus*	*boraceiensis*
SLFT 406	199.15	Cycloramphidae	*Cycloramphus*	*boraceiensis*
SLFT 407	0.00	Cycloramphidae	*Cycloramphus*	*boraceiensis*
SLFT 408	62.21	Cycloramphidae	*Cycloramphus*	*boraceiensis*
SLFT 409	0.00	Hylodidae	*Hylodes*	*asper*
SLFT 410	0.00	Hylodidae	*Hylodes*	*asper*
SLFT 411	0.00	Hylodidae	*Hylodes*	*asper*
SLFT 412	0.00	Hylodidae	*Hylodes*	*asper*
SLFT 413	0.00	Hylodidae	*Hylodes*	*asper*
SLFT 414	0.00	Hylodidae	*Hylodes*	*asper*
SLFT 415	0.00	Hylodidae	*Hylodes*	*asper*
SLFT 416	60.09	Hylodidae	*Hylodes*	*asper*
SLFT 417	178.73	Hylodidae	*Hylodes*	*phyllodes*
SLFT 418	0.00	Hylodidae	*Hylodes*	*phyllodes*
SLFT 419	0.00	Hylodidae	*Hylodes*	*phyllodes*
SLFT 420	0.00	Hylodidae	*Hylodes*	*phyllodes*
SLFT 421	4.01	Hylodidae	*Hylodes*	*phyllodes*
SLFT 422	0.00	Hylodidae	*Hylodes*	*phyllodes*
SLFT 423	0.00	Hylodidae	*Hylodes*	*phyllodes*
SLFT 424	0.00	Hylodidae	*Hylodes*	*phyllodes*
SLFT 425	0.00	Hylodidae	*Hylodes*	*phyllodes*
SLFT 426	0.60	Cycloramphidae	*Cycloramphus*	*boraceiensis*
SLFT 427	0.00	Cycloramphidae	*Cycloramphus*	*boraceiensis*
SLFT 428	0.00	Cycloramphidae	*Cycloramphus*	*boraceiensis*
SLFT 429	0.00	Cycloramphidae	*Cycloramphus*	*boraceiensis*
SLFT 430	0.00	Cycloramphidae	*Cycloramphus*	*boraceiensis*
SLFT 431	4,222.00	Cycloramphidae	*Cycloramphus*	*boraceiensis*
SLFT 432	3,866.77	Hylodidae	*Hylodes*	*phyllodes*
SLFT 433	0.00	Hylodidae	*Hylodes*	*phyllodes*

SLFT 434	0.00	Hylodidae	*Hylodes*	*phyllodes*
SLFT 435	0.00	Hylodidae	*Hylodes*	*phyllodes*
SLFT 436	0.00	Hylodidae	*Hylodes*	*phyllodes*
SLFT 437	0.00	Hylodidae	*Hylodes*	*phyllodes*
SLFT 438	0.00	Hylodidae	*Hylodes*	*phyllodes*
SLFT 439	0.00	Hylodidae	*Hylodes*	*asper*
SLFT 440	0.00	Hylodidae	*Hylodes*	*asper*
SLFT 441	0.00	Hylodidae	*Hylodes*	*asper*
SLFT 442	0.00	Cycloramphidae	*Cycloramphus*	*boraceiensis*
SLFT 443	2.41	Cycloramphidae	*Cycloramphus*	*boraceiensis*
SLFT 444	0.26	Cycloramphidae	*Cycloramphus*	*boraceiensis*
SLFT 445	0.00	Cycloramphidae	*Cycloramphus*	*boraceiensis*
SLFT 446	0.00	Cycloramphidae	*Cycloramphus*	*boraceiensis*
